# Seeking support or avoiding it? A qualitative study of professional psychological help perceptions among university students in Honduras

**DOI:** 10.3389/fpsyg.2025.1565943

**Published:** 2025-06-10

**Authors:** Estyben Rivera, César Barahona-Aguilar, Gamariela Cuevas, Flor Orellana-Moreno, Irma Corrales, Raquel Mejía-Sánchez, Eddy Paz-Maldonado, Miguel Landa-Blanco

**Affiliations:** ^1^School of Psychological Sciences, Faculty of Social Sciences, National Autonomous University of Honduras, Tegucigalpa, Honduras; ^2^Department of Pedagogy and Education Sciences, National Autonomous University of Honduras, Tegucigalpa, Honduras

**Keywords:** mental health, university students, barriers to psychological help, psychological support, psychotherapy, help seeking

## Abstract

University students face significant mental health challenges exacerbated by barriers such as stigma, financial constraints, and limited services. This study examined barriers to seeking psychological help among 23 students from the National Autonomous University of Honduras (UNAH). Participants were recruited through social media, snowball sampling, and campus visits. Using semi-structured interviews, the research explored attitudes, barriers, and perceptions regarding professional psychological help. Interview guides were expert-validated and pilot-tested. Thematic analysis, conducted from a constructivist perspective, identified four themes: motivators, psychotherapy sources, barriers, and facilitators. Findings revealed that while stigma and financial constraints hinder help-seeking, motivators like personal growth and social influences encourage it. The study highlighted the need for accessible mental health services in university settings to address academic and social pressures. Strengthening these services is essential to fostering a supportive environment that promotes student well-being and psychological resilience.

## 1 Introduction

The mental health of young people is a constant concern worldwide (Umubyeyi et al., 2016; Bugtong-Diez, 2020; Nakamura et al., 2022), mainly for universities, as mental health issues are becoming increasingly prevalent and students report significant barriers to accessing professional psychological support (Cage et al., 2020). Key factors hindering psychological help-seeking include age, socioeconomic status, normalized mental strain, public and self-stigma, reliance on independence, and preferring support from friends or family over specialists (Topkaya et al., 2016; Worthley et al., 2017; Cha et al., 2019; Ebert et al., 2019; Ibrahim et al., 2019; Shea et al., 2019; Cage et al., 2020; Landa-Blanco et al., 2021; Gündoğdu et al., 2024). Conversely, students who decide to seek help often encounter overburdened university services and may abandon their attempt to obtain immediate care (Dunley and Papadopoulos, 2019; Farrer et al., 2019; Shi et al., 2019; Sweetman et al., 2021; Yánez, 2022).

Regarding mental health issues, accumulated evidence indicates that stress, anxiety, depression, suicidal ideation or tendencies, and distress are among the most prevalent conditions reported by university students themselves (Grøtan et al., 2019; House et al., 2020; Wang et al., 2020; Lipson et al., 2022).

Universities implement comprehensive strategies in psychological services to support all students, prioritizing vulnerable groups facing unique barriers, including ethnicity, sexual orientation, gender identity, and disability (Fernández-Rodríguez et al., 2019; Liu et al., 2019; Williams et al., 2023; Sigal and Plunkett, 2024; Smith and Hebdon, 2024; Zhang et al., 2024). One alternative proposed in the literature is the adoption of virtual care and intervention mechanisms to support student well-being (Ryan et al., 2010; Chen, 2012; Day et al., 2013; Horgan et al., 2013; Levin et al., 2018; Blair et al., 2024). Similarly, social media platforms serve as critical channels for promoting mental health awareness and facilitating connections with professionals in the field (Landa-Blanco et al., 2024a). However, a significant challenge limiting the effectiveness of virtual care is students’ concerns regarding the credibility and privacy of mobile applications designed for these purposes (Levin et al., 2018).

In universities, advancing toward comprehensive psychological care necessitates considering students’ circumstances and the broader context in which they operate (Jackson, 2018). University students face significant mental health challenges, highlighting the need for research to enhance psychological support services. Honduras addresses structural issues like poverty, exclusion, discrimination, and violence, alongside pronounced mental health stigma and access barriers. These factors emphasize the urgency of interventions (Landa-Blanco et al., 2024c). University students face a range of academic, social, and economic pressures, and their mental health needs may go unmet due to cultural, logistical, or financial barriers. A study conducted among students at the National Autonomous University of Honduras (UNAH) revealed that poor sleep quality and low self-esteem are associated with greater barriers to seeking psychological help (Landa-Blanco et al., 2024b).

This study explored the perceived barriers students at the UNAH face when seeking professional psychological help. Using a qualitative, phenomenological approach (Creswell and Poth, 2018), it examined the experiences of students who have accessed mental health services as well as those who have not. The goal was to identify key factors shaping students’ attitudes and behaviors toward seeking psychological support. Specific thematic areas included motivations, facilitators, barriers, and the perceived availability of mental health resources.

## 2 Methods

### 2.1 Participants

The primary sample consisted of 23 undergraduate students registered in the UNAH, with 48% (*n* = 11) identifying as female and 52% (*n* = 12) as male. Their ages ranged between 18 and 26 years. Participants were recruited through a combination of social media outreach, snowball sampling, and campus visits. The initial aim was to achieve a balanced sample based on sex and prior experience with professional psychological help, including both students who had sought such help (*n* = 10) and those who had not (*n* = 10). However, all ten participants without prior experience expressed positive attitudes toward seeking psychological help. To ensure a broader range of perspectives, additional participants explicitly opposed to seeking professional psychological help were recruited. Despite these targeted efforts, only three additional participants meeting this criterion were available for inclusion (see Table 1). Coding and data collection were done in parallel to assess theoretical saturation. The number of new codes generated in every interview was recorded. Although reasonable saturation was achieved by the 12th interview (91% of codes), 11 more interviews were collected to ensure it was properly represented.

**Table 1:** Characteristics of the participants and thematic codes.

Interview	Sex	Age	Career	Characteristic	New codes
Interview 1	Female	19	Psychology	Assisted	7
Interview 2	Male	20	Finance	Not assisted	11
Interview 3	Female	20	Psychology	Not assisted	9
Interview 4	Male	21	Psychology	Not assisted	3
Interview 5	Female	18	Psychology	Not assisted	9
Interview 6	Male	26	Laws	Assisted	4
Interview 7	Female	20	Psychology	Assisted	5
Interview 8	Female	19	Dentistry	Assisted	1
Interview 9	Male	24	Literature	Assisted	1
Interview 10	Female	21	Psychology	Not assisted	0
Interview 11	Male	22	Architecture	Not assisted	0
Interview 12	Male	19	Engineering	Not assisted and opposed it	0
Interview 13	Male	19	International Commerce	Not assisted	1
Interview 14	Female	23	Programming	Not assisted	1
Interview 15	Female	19	Psychology	Not assisted	2
Interview 16	Male	20	Communication sciences	Not assisted	0
Interview 17	Female	20	Psychology	Assisted	0
Interview 18	Male	19	Psychology	Assisted	1
Interview 19	Male	24	Engineering	Not assisted and opposed it	0
Interview 20	Male	23	Engineering	Not assisted and opposed it	0
Interview 21	Male	20	Psychology	Assisted	0
Interview 22	Female	22	Biology	Assisted	0
Interview 23	Female	21	Engineering	Assisted	0

“Not assisted” refers to individuals who have not received professional psychological help, while “assisted” refers to those who have. “Not assisted and opposed” describes individuals who have neither received professional psychological help nor support the idea of receiving it.

To triangulate the data, a discussion group was conducted; five clinical psychologists participated in the discussion. These secondary participants were recruited through the alumni bank of the Master’s Degree in Clinical Psychology at UNAH.

The sample size in this study is considered adequate based on the principles of information power, which suggests that the more information the sample holds relevant to the study aims, the fewer participants are required (Malterud et al., 2016). In this case, the aim was specific and focused: to explore perceived barriers to seeking professional psychological help among university students in Honduras. The sample was also highly specific, consisting of students from a single university, selected based on their experiences with or attitudes toward psychological help-seeking. The study was informed by established theory and prior research on mental health and help-seeking behaviors, which shaped both the interview guides and the analytical approach. The quality of dialogue was ensured through expert-validated, pilot-tested interview guides, in-depth interviews, and the use of member checking. Finally, the thematic analysis employed was rigorous and iterative, including multiple rounds of coding, consensus-building, and triangulation with external experts. Together, these factors support the adequacy of the sample size, and data saturation was confirmed after the 12th interview, with additional interviews conducted to ensure analytical richness.

This study was conducted at UNAH, the country’s largest public university. The research took place within the broader context of a developing nation facing significant structural barriers to mental health care, including stigma, limited access to services, and economic hardship. UNAH students often navigate high academic demands while contending with sociocultural pressures discouraging help-seeking, particularly among men (Landa-Blanco et al., 2024a; Landa-Blanco et al., 2024b; Landa-Blanco et al., 2024c; Landa-Blanco and Mejía Sánchez, 2025). The university setting was chosen due to its diverse student population and because it represents a critical context in which mental health challenges are both prevalent and insufficiently addressed. This setting provided a relevant and rich environment for exploring the perceptions and barriers related to professional psychological help among emerging adults in Honduras.

### 2.2 Data collection techniques

Two semi-structured interview guides were developed: one for students with prior experience receiving professional psychological help and one for students without such experience. Additionally, a focus group guide was developed for clinical psychologists to gather complementary insights and support data triangulation (see Supplementary material). These guides were then validated by a panel of three experts, comprising a qualitative research expert and two clinical psychologists with relevant research backgrounds. The experts evaluated the questions’ relevance, pertinence, and overall structure, offering suggestions for deletions, modifications, or additions. Following the expert feedback, the revised interview guides were subjected to a pilot testing phase using cognitive interviews with two university students. During these cognitive interviews, participants were asked to respond to the questions and provide feedback on their understanding of the wording, the flow of the questions, and any ambiguities they encountered. This process helped to identify potential misunderstandings or difficulties in interpreting the questions. As a result, further adjustments were made to improve question clarity, refine language, and enhance the overall structure of the guides, ensuring that they were accessible and relevant to the target population.

The interview guide varied depending on whether the respondent had previously sought professional psychological help. Some of the questions included: “What do you think your family and friends think about your decision to attend therapy?,” “Have you faced any issues that made it difficult to continue attending therapy?,” “What are your thoughts on professional psychological help and therapy in general?,” “What do you think are the main reasons some people seek professional psychological help?,” “Do you have any fears or concerns about attending professional psychological help?,” among others.

### 2.3 Procedures and data analysis

This study was approved by the Research Ethics Committee of the Faculty of Social Sciences (UNAH), under registration number CEIFCS-2023-P2, as part of a broader study of psychosocial wellbeing in university students. The research began with a comprehensive literature review to develop a theoretical framework and identify gaps in understanding students’ perceptions of professional psychological help. Two semi-structured interview guides were crafted to address participants’ perspectives with and without prior experience seeking such help. These guides underwent rigorous validation by a panel of three experts, who assessed their relevance, clarity, and comprehensiveness. Following expert feedback, the guides were refined through cognitive interviews with two university students to resolve any issues with wording or structure, ensuring they were well-aligned with the research objectives.

Data collection involved interviews with participants in person or online using Google Meet. These interviews were conducted between June and July of 2024. Each interview was recorded and transcribed for analysis. All participants signed an informed consent. After each interview, the interviewers conducted a “member check.” During this process, they summarized the key points of the interview and presented this summary to the participant for validation. Participants were invited to provide feedback on the accuracy of the summary and the thematic interpretation. This step enhanced credibility by ensuring the findings accurately reflected the participants’ perspectives and experiences. It allowed participants to correct any inaccuracies and offer additional insights, thereby increasing the trustworthiness of the data. During data collection, all interviewers took detailed field notes, which were scanned and attached to each transcript.

The research team consisted of clinical psychologists, faculty members, and advanced psychology students trained in qualitative research and mental health. Their academic backgrounds and clinical experience enriched the study’s design, interview techniques, and interpretation of data. All interviewers were affiliated with the same university as the participants, which may have influenced participant openness positively due to shared institutional and cultural understanding. At the same time, the team remained reflexive throughout the research process, acknowledging potential biases stemming from their professional commitment to mental health advocacy. To mitigate these influences, the team implemented expert validation of instruments, member checks, peer debriefings, and consensus-based coding. These strategies supported transparency and minimized the impact of presuppositions on data collection and interpretation, enhancing the credibility and transferability of the findings.

The data analysis for this study employed thematic analysis, a qualitative method ideal for identifying patterns within complex data. The process consisted of several stages: initial read-through, initial coding, focused coding, axial coding, and integration. The initial read-through was followed by coding, where the data was categorized into preliminary codes to identify emerging concepts. Focused coding organized these codes into broader themes. Axial coding explored relationships between these themes, and integration synthesized the themes into a cohesive narrative of the study’s findings. This analysis followed a constructivist approach, recognizing that participants’ experiences and meanings are shaped by their social and cultural contexts. The coding process involved multiple rounds of discussion and consensus-building among researchers, ensuring the participants’ perspectives were accurately captured.

To ensure the findings’ dependability, four participants were re-interviewed after the analysis to confirm the relevance of the themes and codes. Their feedback refined and validated the findings. Additionally, the findings were reviewed by a discussion group of five clinical psychologists, adding an external check on the data and minimizing researcher bias. This expert validation, alongside member checks, ensured the findings were grounded in the data and expert knowledge. An audit trail was maintained throughout the study, documenting each research step, including data collection methods, coding decisions, and thematic development. This documentation ensured transparency, rigor, and trustworthiness in the research process.

The research also maintained credibility, dependability, and confirmability through member checks, iterative validation, and expert review. All interviews were conducted in Spanish, and the translations of selected phrases were reviewed and validated by independent researchers and a certified English professor to ensure accuracy. This comprehensive approach strengthened the study’s rigor and ensured the findings accurately reflected participants’ experiences.

## 3 Results

**Figure 1:**
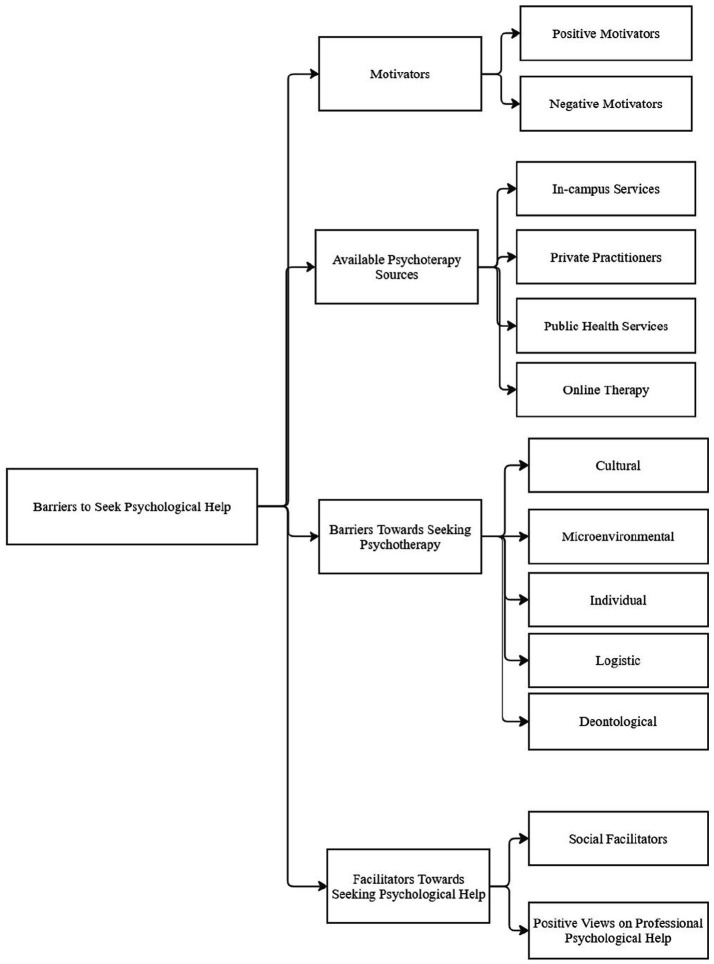
Main themes related to the attitudes and experiences of seeking professional psychological help.

This research focused on four main themes, from which subthemes and their respective codes emerged through the interviews. The first overarching theme was Motivators, which branched into Positive Motivators and Negative Motivators. The second theme, Available Psychotherapy Sources, included the In-campus Services, Private Practitioners, Public Health Services, and Online Therapy subthemes. The third theme, Barriers to Seeking Psychotherapy, encompassed Cultural factors, Microenvironmental factors, Individual factors, Logistic factors, and Professional Mistrust. Finally, the fourth theme, Facilitators Towards Seeking Psychological Help, was divided into Social Facilitators and Positive Views on Professional Psychological Help (see Figure 1 and Table 2).

**Table 2:** Themes, subthemes and codes related to the attitudes and experiences of seeking professional psychological help.

Theme	Subtheme	Code	Description	Sample quote
Available psychotherapy sources		In-campus services	Therapy services provided by the university.	“If I want free therapy, I go to the university’s [therapy service], but as I mentioned, there’s no space. As for the cost, I understand they are around 700 per session. They’re expensive.” - Interview 19
		Public health services	Therapy services offered by government-funded health centers or programs.	“A lot, because for example the health centers that offer psychological care tend to have it too restricted, so I do not feel like there would be real progress, so the only option in that case would be private, and it’s very difficult to find a psychologist who inspires confidence.” - Interview 20
		Non-profit organizations	Therapy services provided by non-profit organizations.	“In this case, I can enjoy psychological therapy for free. In reality, I’ve never had to pay for therapy as such. Initially, I was in a non-profit organization. “- Interview 6
		Private practice	Therapy services provided by individual therapists.	“I think it’s not very accessible. I feel that, in general, the prices, in terms of cost, I think a private appointment, at least one appointment, I think they are about 900 lempiras, I’m not sure, but I do feel that it’s not very accessible.” - Interview 23
		Online therapy	Therapy sessions conducted remotely via the internet.	“Online therapy is a way for the person to communicate with others. Technology is practically something that has benefited the world a lot, because there are people who for X or Y reason have not wanted to be seen as a person, if a psychologist, they have not wanted to be seen buying things so they do not know they have a problem. Something beneficial is that, in their privacy, they can do online therapy, talk to a professional, discuss the issues, and these are things that remain totally between them and the person who saw them.” - Interview 13
Barriers towards seeking psychotherapy	Cultural barriers	Stigma	Refers to the negative perception and discrimination associated with seeking psychological help, often leading to social rejection.	“I faced many challenges when considering starting therapy, like society’s stigmatization and derogatory comments about the competence of those who receive psychological treatment.” - Interview 6 “They do not believe in it. I mean, if you talk to them about therapy, they just mock it because they think it’s nonsense, a waste of time.” - Interview 13 “Yes, at school, where I was, the kids were not very educated about it. So, because they lacked education, they behaved very stupidly, like mocking, saying ‘oh, you are crazy,’ and things like that.” - Interview 10
		Lack of perceived importance of mental health	Indicates the undervaluation of mental health and the belief that psychological services are unnecessary or less important.	“Yes, I think more campaigns should be conducted to demystify and value psychology. Many people still undervalue psychology and do not recognize its importance in mental health.” - Interview 5 “I think the biggest difficulty is that psychology is not given the importance it deserves.” - Interview 17
		Male chauvinism	Describes how traditional gender roles and societal expectations discourage men from seeking psychological help, associating vulnerability with weakness.	“Hmm, ignorance. In our country, boys are generally told, ‘You should not cry, you are a tough guy, do not do it.’ Or they see going to a psychologist as foolish, thinking they can fix it by punishing or hitting the boy or girl. And that’s why you are wrong; things were worse before us, and look, here we are. Although now, well, I have not seen many ads, but, for example, Ciudad Mujer promotes it a lot. And if I’m not mistaken, they also offer that service there. But the problem is that it’s targeted only at women. And men, in my opinion, also need that help, that attention. Because no, not for them. So, they are being undervalued because both men and women need help. Although men always make up excuses not to go. But I think there should be a place for their attention, whether it’s psychological.” - Interview 14 “A culture where a man is seen as homosexual, as gay, if he cries. You grow up with those silly ideas in your mind sometimes. Things like that. Yes, as I said, in this country, some people have conservative minds, especially in rural areas because the level of education does not allow them to see other perspectives on these issues. You’ll always hear that stupid comment that men should not cry. I heard it from a mother who is my neighbor, where I come from, telling her younger son that crying is only for girls.” - Interview 16
		Physical punishment	Involves the use of physical discipline as a means to correct behavior.	“Those beatings they gave me or those talks they had with me did not help. They saw there was no improvement. So, the night before my first appointment, both of them told me that I was going to attend a psychological process, to receive psychological assistance.” - Interview 18
	Microenvironmental barriers	Family disapproval of therapy	Refers to the negative attitudes and lack of support from family members towards therapy, often due to conservative beliefs or misunderstandings about its value.	“On my mom’s side of the family, they are very conservative. They do not believe in it. I mean, if you talk to them about therapy, they just mock it because they think it’s nonsense, a waste of time.” - Interview 13
		Friends disapprove of therapy	Describes the lack of support or outright dismissal of therapy by friends, who may believe that psychological issues are not real or that therapy is unnecessary.	“My friends said that it did not exist, that it was just in our heads.” - Interview 9
		Lack of information	Refers to the scarcity of public knowledge about mental health services, making it difficult for people to understand where to seek help or the importance of therapy.	“Yes, and that’s what makes it so inaccessible, because there is not much public information. It’s not like when someone has a heart problem and knows where to go, but when it comes to mental health, it’s still not clear where to turn. I think the biggest difficulty is that psychology is not given the importance it deserves.” - Interview 17
	Individual barriers	Resistance to open up	Refers to individuals’ difficulty in expressing their thoughts and feelings openly, often due to lack of experience or discomfort in vulnerability.	“It would mostly be the aspect that I’ve never really, honestly opened up to a person. So, I would not really know, and that’s a difficulty I have; I’m not very expressive. So, I would not know what words to use to express myself in therapy.” - Interview 13
		Fear of going to therapy	Describes the apprehension or anxiety about attending therapy, often stemming from uncertainty or external pressures	“Yes, I was scared. And as I mentioned, it wasn’t my idea; it was my mom’s.” - Interview 8
		Fear of being judged	Refers to the concern that others may criticize or think poorly of someone for attending therapy, leading to feelings of embarrassment or reluctance to seek help.	“There are people who are greatly influenced by what others will say because they fear being judged. They might think, ‘Am I different from those people just because I go to therapy?’ And they start saying I’m crazy. Maybe it’s the fear of opening up to someone I do not know, who could judge me. Fear of feeling judged.” - Interview 15
		Therapy as ineffective	Reflects the belief that therapy will not help or that it may even worsen one’s mental state, often based on negative perceptions or experiences shared by others.	“People react by saying I do not need to go to that crazy place. I do not need someone to make me crazier. Comments like that, pessimistic ones. Because there are cases nowadays where they tell you that when you go to the psychologist, you are going to a madhouse. That it’s useless, you do not need it.” - Interview 18
		Psychologist as a stranger	Describes the discomfort some individuals feel in sharing personal issues with a therapist they barely know, leading to hesitation or incomplete disclosure.	“Well, for me, it’s generally easier to say the things I think, but I feel kind of strange. But still, sometimes it can be uncomfortable to come and expose your entire self to someone else, to someone you have just met or do not know at all.” - Interview 14
		Therapy as unnecessary	Represents the belief that therapy is not needed, often due to lack of understanding, awareness, or the perception that one can handle problems independently.	“The truth is, as someone who wasn’t well-informed and had not experienced things, I do not know. I did feel like it was unnecessary or that one could solve their problems without therapy. I was very ignorant about it.” - Interview 8
		Autonomous conflict management	Reflects the desire to handle personal issues independently, without relying on therapy or others, due to a preference for self-sufficiency.	“A little uncomfortable, honestly, because I feel like I might need it, but at the same time, I do not want to depend on someone else. I do not want to always depend on something else; I want to solve my problems on my own.” - Interview 20
		Role confusion between psychologists and psychiatrists	Refers to the common misunderstanding where people confuse the roles and functions of psychologists and psychiatrists, often due to lack of information.	“So, I feel like most people, in general, have the ideology of confusing what psychiatrists are with psychologists. Due to a lack of information.” - Interview 13
		Hopelessness	Reflects the belief that therapy or psychological help will not make a difference in one’s situation, leading to a sense of despair or resignation.	“No, it’s not important to go to a psychologist, nothing will help me.” - Interview 15
Facilitators toward seeking psychological help	Logistical barriers	Financial constraints	Refers to the economic barriers that prevent individuals from accessing psychological services, such as the cost of therapy, transportation, or other associated expenses.	“I think it’s not very accessible. For people with fewer resources, it’s a bit more difficult to attend, even if the services are free, because not all places offer free care. And so there can be several factors preventing those people from reaching that service, like transportation costs.” - Interview 15
		Location	This refers to the challenges related to the physical location of mental health services, including distance, transportation issues, and the service provider’s accessibility.	“Not knowing about them, I think the first thing would be the cost, because you do not know how much the person will charge you, where you are going to go, because you do not just have to schedule the appointment, you also have to get there, and you do not know if it’s close to where you live, what time you have to leave, if you’ll get stuck in traffic, if you’ll arrive on time, and if you do not arrive on time, they reschedule you, and you have to pay for that.” - Interview 14
		Waitlists	Describes the delays in receiving psychological care due to long waiting periods, which can be particularly problematic when immediate help is needed.	“Time. Money. Well, regarding money, it could be, but it’s not that much, because if there’s free help. But, for example, here at the university, there’s the CAPS, so to speak. You have to sign up on a list. Let us say you go there because you need quick help, so to speak. And they tell you, no, you have to wait on the waiting list, and let us see, at any moment we’ll call you if a slot opens up. What if that slot opens up in 3 months? So those would be the reasons.” - Interview 15
		Remittance	Refers to the requirement for a referral from a medical doctor before accessing psychological services, often seen in public health systems.	“And sometimes, maybe in public places, going to a psychology consultation, they do not accept you unless it’s with a medical referral, you could say.” - Interview 5
		Time management	Refers to the difficulty of aligning therapy schedules with personal, academic, or professional responsibilities, which can hinder consistent attendance.	“A problem I faced was the difficulty of adjusting therapy schedules with my university schedule, which sometimes made it hard for me to attend on time.” - Interview 7
	Deontological barriers	Lack of professionalism	Refers to situations where psychologists do not provide adequate care or attention to their patients, prioritizing financial gain over patient well-being.	“Because there are some psychologists who do not give importance to their patients and just take their money, which is wrong.” - Interview 18
		Lack of Rapport	Describes the discomfort and difficulty in establishing a positive and trusting relationship with a therapist, which can affect the effectiveness of therapy.	“At first, it was super uncomfortable. You do not know what your psychologist will be like. On one occasion, I had a very kind lady, but on another, I got a very unpleasant lady.” - Interview 8
		Confidentiality concerns	Reflects the fear that therapists might share personal information with others, such as parents, without the patient’s consent, leading to distrust in therapy.	“But there’s the concern about whether they might tell something to our parents without asking us first.” - Interview 8
	Social facilitators	Positive Experiences reported online	Refers to the influence of positive accounts of therapy shared on social media or other online platforms, which can shape individuals’ perceptions of psychology and therapy.	“I feel that other people who have gone to therapy, from videos I’ve seen on TikTok, videos I’ve seen on social media in general, and people who have had good experiences in therapy, have greatly influenced my thoughts about psychology.” - Interview 12
		Positive experiences reported by friends and family	Describes how the positive outcomes of therapy experienced by friends and family can encourage others to view therapy favorably and consider it a valuable resource.	“With some friends who have overcome their problems thanks to therapy, close people I’ve seen, that’s why I consider it good.” - Interview 2 “Very beneficial because, in my case, I had a family member who suffered from depression, and thanks to therapy, they gradually overcame the problem because they reached a point where they wanted to commit suicide. So, I’m more than aware that therapy is something that greatly benefits people who really suffer from different, I do not know if we should call them disorders, but rather problems they may face in their life.” - Interview 13
		Family Support for Therapy	Refers to the encouragement and positive attitude towards therapy within a family, which can make it easier for individuals to seek and continue with therapy.	“I’ve always been very open about therapy, and my family environment has also been very receptive. I was never made to feel that going to therapy meant I was ‘crazy.’“- Interview 7 “My friends said that it did not exist, that it was just in our heads. But my family did see it as a good decision.” - Interview 9
		Therapy normalized	Describes how therapy is viewed as a normal and acceptable part of life, particularly within certain social or cultural groups, reducing stigma and encouraging its use.	“Honestly, no. As I mentioned, I’m not surrounded by people who see it negatively. I’m surrounded by people who normalize it. Maybe some have doubts, but I just explain it to them, and they no longer hold any prejudice or anything.” - Interview 5 “
	Positive views on professional psychological help	Psychotherapy perceived effectiveness	Refers to the belief that psychological therapy is beneficial and necessary, and that changing societal attitudes towards therapy is important for mental health.	“Psychological therapy is something positive, necessary, and something that people in this country need to change their viewpoint on and see it as something positive and necessary. I’ll repeat it in two words because they motivate me to seek therapy: it’s something positive and necessary.” - Interview 16
		Psychologists as trained professionals	Describes the recognition of psychologists as highly trained professionals capable of helping individuals work through traumas or problems, leading to potential solutions.	“It’s something that greatly benefits people because, unlike me, there are people I know who have had various traumas or problems in their lives and have not had anyone who, so to speak, is more trained in the field to discuss the problem and arrive at a possible solution.” - Interview 13

### 3.1 Theme 1: motivators to seek psychological help

Participants identified both positive and negative motivators for seeking therapy. Positive motivators included the desire for self-understanding, emotional balance, and overall well-being. For example, one participant described therapy as a means to “discover qualities that one may be unaware of in difficult situations” (Interview 20). Another emphasized the importance of “emotional balance,” stating that it allows one to “feel better” (Interview 3).

Participants also identified negative motivators, which were often reactive, driven by psychological distress or unresolved emotional issues. Participants mentioned challenges such as difficulty regulating emotions, low self-esteem, anxiety, depression, grief, trauma, and family conflicts as triggers for seeking therapy. The intensity and duration of these symptoms were key factors in motivating individuals to seek help. One participant acknowledged their tendency to delay therapy, stating, “I go when I’m already feeling really bad” (Interview 8). Another reflected on the cumulative effect of unresolved issues, saying, “Over time, all those effects are felt” (Interview 5). The need for emotional release and catharsis was also noted, with one interviewee explaining, “Therapy helps you vent, resolve internal problems, and treat hidden traumas” (Interview 5).

Additionally, the triangulation group reported common issues in therapy consultations, including anxiety, marital problems, depression, grief, suicidal ideation, and behavioral issues, among others. This supports the notion that negative motivators play a significant role in therapy-seeking behavior, as personal challenges—both positive and negative—serve as catalysts for seeking help.

### 3.2 Theme 2: available psychotherapy sources

In exploring the barriers to seeking psychological help, interviewees highlighted various sources of psychotherapy, each with distinct challenges and advantages. Participants identified five primary sources: in-campus services, non-profit organizations, public health services, private practitioners, and online therapy.

Likewise, the triangulation group stated, “The pandemic has made virtual therapy more common, which makes access easier for some, but also presents challenges for those who do not have access to technology.” This aligns with the interviews, which emphasize that online psychological services have emerged as a viable option, benefiting some individuals by providing access to psychological support.

### 3.3 Theme 3: barriers to seeking psychotherapy

The barriers to seeking professional psychological help were classified into five themes: Cultural, Microenvironmental, Individual, Logistic, and Deontological. Participants in this study identified several key cultural factors that hinder individuals from pursuing therapy, including stigma, the lack of perceived importance of mental health, male chauvinism (machismo), and the prevalence of physical punishment as a disciplinary method.

Social disapproval and lack of awareness emerge as significant barriers to seeking psychological help. Participants in this study highlighted the negative impact of family and friends’ disapproval of therapy, often fueled by conservative beliefs and misunderstandings about mental health. Additionally, a pervasive lack of information about mental health services further exacerbates these challenges, leaving individuals unsure of where to turn or undervaluing the importance of therapy.

Individual barriers to seeking psychological help can profoundly influence a person’s willingness or ability to engage in therapy. Participants in this study identified several key individual barriers, including resistance to opening up, fear of therapy, concerns about being judged, skepticism about the effectiveness of therapy, discomfort with sharing personal issues with a stranger, and a belief in the sufficiency of self-reliance. Additionally, confusion about the roles of psychologists and psychiatrists and feelings of hopelessness further complicate the decision to seek professional help.

Logistic barriers significantly impact individuals’ ability to access psychological services. Participants in this study highlighted several critical logistical challenges, including financial constraints, location issues, long waitlists, the need for medical referrals, and time management difficulties.

Trust and professionalism are critical factors that can make or break the therapeutic experience, yet participants in this study reported significant concerns in these areas. Issues such as a lack of professionalism, where psychologists are perceived to prioritize financial gain over patient care, and difficulty in establishing rapport, where patients struggle to connect with their therapists, were commonly cited.

The triangulation group identified several barriers to seeking psychological help, including persistent stigmas, such as the fear of being labeled “crazy,” and concerns about confidentiality, especially in sensitive cases like HIV. Despite some progress, stigma and the perception of therapy as unnecessary remain widespread. Bad past experiences with therapists often deter individuals from seeking help. Interviewees echoed these sentiments, noting that cultural stigma and derogatory remarks about therapy seekers reinforce negative perceptions. The microenvironment also plays a role, with limited therapy access and a lack of commitment affecting the therapeutic process. Fear of diagnosis, especially among parents, and the absence of psychoeducation were significant barriers. Resistance to change and a lack of commitment were also noted. Logistic factors, such as costs and therapy location, were also highlighted, with many facing challenges due to limited access and high treatment costs. These findings were consistent with interviewees’ statements about the financial burden and difficulty accessing therapy services. Additionally, due to resource shortages, long waiting lists in public health systems were cited as a significant barrier. The triangulation group noted the lack of investment in prevention and resource limitations, aligning with interviewees’ experiences, which prevent individuals from seeking or continuing therapy.

### 3.4 Theme 4: facilitators towards seeking psychological help

Participants in this study highlighted how positive experiences shared online and those reported by friends and family have significantly influenced their perceptions of therapy. They also expressed a strong belief in the effectiveness of psychological therapy, viewing it as not only beneficial but also beneficial and essential for mental well-being. This perception is reinforced by recognizing psychologists as highly trained professionals capable of helping individuals navigate complex traumas and problems.

The triangulation group stated, “Establishing a good connection from the first session is essential. Patients come with expectations and fears, and creating a safe and trusting environment is key for them to feel comfortable and continue with therapy. The structure of the therapy and clarity in the process are important for the success of the treatment.” This is consistent with what the interviewees expressed, stating that when therapy is perceived as effective and psychologists are seen as competent professionals, it fosters a positive view of professional psychological help.

### 3.5 Member check

After each interview, participants were asked to confirm the accuracy of a summary of the key points reflecting their narratives. Once all interviews were analyzed and a draft of the results was completed, four randomly selected respondents participated in a final member check. During this stage, the aggregated results, including themes, subthemes, codes, and interview excerpts, were presented to the participants. They were asked to comment on whether the findings aligned with their perceptions.

The feedback from the final member check supported the validity and relevance of the study’s findings. One participant agreed with the research, emphasizing the importance of emotional support, trust-building with the psychologist, and progress observed over time. They also highlighted barriers such as social judgment, lack of information about therapy, and issues related to cost and accessibility as significant obstacles many people face when seeking therapy (Interview 2). Another participant confirmed the consistency of the barriers across interviews, noting that although the responses varied, the presence of common barriers, such as stigma, underscored their significance (Interview 13). A third respondent praised the research for being comprehensive and capturing a wide range of difficulties encountered when seeking therapy, including therapy protocols, symptoms, and common societal challenges (Interview 9). Lastly, another participant affirmed that the results accurately reflected the factors influencing therapy-seeking decisions, noting the coherence between their own experiences and the study’s conclusions (Interview 22).

The final member check reinforced the credibility and reliability of the qualitative findings. By presenting the participants with the themes and interview excerpts, the researchers allowed respondents to directly assess the alignment between their personal experiences and the study’s outcomes. The feedback indicated that the findings were consistent with the participants’ perceptions, ensuring their voices were faithfully represented in the research. The validation process enhanced study rigor, ensuring trustworthiness and authenticity. Participants confirmed the results accurately reflected their experiences and barriers in seeking psychological help, strengthening the study’s credibility.

## 4 Discussion

This article explored the perceived barriers faced by university students enrolled at the National Autonomous University of Honduras when seeking professional psychological help. A qualitative study was conducted to capture the perspectives. The findings focused on four key aspects: (1) Motivators for seeking psychological help, (2) Psychotherapy sources, (3) Barriers to seeking help, and (4) Facilitators of seeking psychological help.

This study underscores positive motivators like self-awareness, growth, and emotional balance, alongside adverse motivators such as emotional distress, anxiety, depression, and low self-esteem, as key elements influencing the pursuit of psychological support. The interaction between positive and adverse motivators significantly shapes the decision to seek psychological help, ultimately fostering personal development, emotional relief, and enhanced self-understanding. These findings align with existing research, which highlights the importance of fulfilling basic psychological needs and strengthening self-regulation as central factors in motivating individuals to seek psychological assistance (Gündoğdu et al., 2024; Worthley et al., 2017).

The physical environments where psychological therapy occurs play a pivotal role in addressing the increasing demand for mental health services (Jackson, 2018; Munyaradzi and Addae, 2019). While university campus services are intended to be primary sources of support, they are often overwhelmed by demand and relatively expensive for student programs (Farrer et al., 2019). Public health services face long waiting lists and reliability issues, while private healthcare offers quality services but is financially inaccessible. In Latin America, limited hospital mental health care disproportionately impacts low-income groups (Fernández-Rodríguez et al., 2019; Liu et al., 2019; Yánez, 2022; Williams et al., 2023; Sigal and Plunkett, 2024). Online therapy has emerged as a valuable alternative, providing local accessibility and enhancing trust through the convenience and anonymity of virtual sessions. Online interactions significantly influence positive attitudes toward remote therapy, making this method a practical, convenient, and cost-effective option for individuals unable to attend in-person sessions (Ryan et al., 2010; Day et al., 2013; Horgan et al., 2013; Levin et al., 2018; Lancia et al., 2023; Blair et al., 2024).

Barriers to seeking psychological support are multifaceted, with stigma as a key obstacle. Cultural norms perpetuate misconceptions, such as associating mental health care with being “crazy,” fostering fear of judgment. This stigma diminishes the perceived value of services, discouraging individuals from seeking help and hindering access to care and treatment continuity (Topkaya et al., 2016; Cha et al., 2019; Ebert et al., 2019; Ibrahim et al., 2019; Shea et al., 2019; Cage et al., 2020). Cultural beliefs exacerbate barriers to seeking psychological support, particularly those that normalize physical punishment as a method of behavior regulation or advocate enduring adversity instead of pursuing emotional assistance; these attitudes are closely linked to male chauvinism, which often frames men seeking psychological help as a sign of weakness. Such norms, contribute to greater reluctance among men to seek help (Smith and Hebdon, 2024).

An additional obstacle to seeking psychological help is rooted in the individual’s microenvironment, where prevailing ideas, prejudices, and a lack of psychoeducation can present considerable challenges (Zhang et al., 2024). A microenvironment with a negative perception of psychological support may spread misinformation, leading to confusion and uncertainty about seeking help. This, in turn, can foster feelings of isolation and insecurity, making it even more challenging for individuals to seek help (Landa-Blanco et al., 2024a; Landa-Blanco et al., 2024c).

Individual barriers significantly influence the decision to seek psychological help, as they are deeply rooted in personal beliefs and emotions, making them particularly difficult to overcome (Grøtan et al., 2019; Wang et al., 2020; Lipson et al., 2022). These barriers not only obstruct the pursuit of psychological support but also affect an individual’s commitment to therapy. Key factors include reluctance to disclose personal issues, particularly to a stranger such as a psychologist, fear of being judged during therapy sessions, and skepticism about the effectiveness or necessity of therapy. Moreover, this resistance is frequently linked to a conflict with personal autonomy (Sweetman et al., 2021). In line with previous literature, doubts about the effectiveness of therapy services, stigma, and the fear of judgment underscore the complexity of individual perceptions; these factors reinforce a sense of self-sufficiency that deters individuals from seeking professional help (Dunley and Papadopoulos, 2019).

Logistic barriers are critical factors that significantly impact individuals’ ability to seek psychological help. These barriers complicate access to therapy and disrupt the consistency of attending sessions. This study highlighted several logistical challenges, including financial constraints, geographic limitations, long waiting lists, and the time commitment required for psychological appointments. Such logistical challenges hinder individuals from accessing formal mental health care (Umubyeyi et al., 2016; House et al., 2020).

A lack of trust in the mental health professional represents another significant barrier to seeking psychological help; perceived issues such as unprofessional conduct, challenges in building rapport, and concerns about confidentiality can lead to negative experiences. Distrust in psychologists hinders undergraduate students from pursuing psychological assistance, as it raises doubts about the effectiveness of treatment (Chen, 2012).

This study highlighted two primary factors that facilitate the process of seeking psychological help: social facilitators and a positive perception of professional psychological support. Social influence, such as shared positive experiences within families, among friends, or through online platforms, plays a critical role in normalizing therapy and easing the decision to seek assistance. Consistent with existing literature, social support is a key motivator for individuals to pursue, remain engaged in, and commit to professional psychological services (Bugtong-Diez, 2020; Nakamura et al., 2022).

In conclusion, this study identifies barriers to seeking psychological help, including stigma, internalized beliefs, and social factors, which often lead individuals to rely on self-help or ignore their distress. However, motivators like self-awareness, personal growth, and social influences encourage seeking therapy. Accessing professional support is essential for mental health improvement, especially for students facing academic and social stress.

The study’s limitations include its focus on students and difficulties in contacting those opposed to seeking help. Qualitative research limits generalizability but provides valuable contextual insights. Future research could explore additional social factors, assess the effectiveness of therapeutic interventions, and investigate the role of psychological services, policies, and interdisciplinary teams in supporting students.

## Data Availability

The raw data supporting the conclusions of this article will be made available by the authors, without undue reservation.
