# Supplementary material for: Seeking support or avoiding it? A qualitative study of professional psychological help perceptions among university students in Honduras
Source: Front Psychol. 2025 Jun 10;16:1565943. doi: 10.3389/fpsyg.2025.1565943 (PMC12186536; doi:10.3389/fpsyg.2025.1565943)
Supplement: Supplementary file 1 [file Data_Sheet_1.pdf]

Supplementary Material

Interview Guide for Individuals Who Have Not Attended Therapy

Question number	Question
1	Have you ever considered seeking psychological help or therapy? Why or why not? (¿Alguna vez has considerado buscar ayuda psicológica o terapia? ¿Por qué o por qué no?)
2	What are your thoughts on psychological help and therapy in general? (¿Cuáles son tus pensamientos sobre la ayuda psicológica y la terapia en general?)
3	Do you believe psychological therapy can be beneficial or not? Why or why not? (¿Crees que la terapia psicológica puede ser beneficiosa o no? ¿Por qué o por qué no?)
4	What do you think are the main reasons why some people seek psychological therapy? (¿Cuáles crees que son las principales razones por las que algunas personas buscan terapia psicológica?)
5	What are the specific reasons why you have not sought psychological therapy? (¿Cuáles son las razones específicas por las que no has buscado terapia psicológica?)
6	Have you had any experiences that influenced your view of psychological therapy? (¿Has tenido alguna experiencia que haya influido en tu visión sobre la terapia psicológica?)
7	Do you have any fears or concerns about attending psychological therapy? (¿Tienes algún miedo o preocupación sobre asistir a terapia psicológica?)
8	How do you usually cope with emotional or psychological challenges? (¿Cómo sueles enfrentar los desafíos emocionales o psicológicos?)
9	What do you think your family and friends think about psychological therapy? (¿Qué crees que opinan tu familia y amigos de la terapia psicológica?)
10	Have you ever felt judged for considering psychological therapy? (¿Alguna vez te has sentido juzgado por considerar la terapia psicológica?)
11	How does culture influence your decision about psychological therapy? (¿Cómo impacta la cultura en tu decisión sobre la terapia psicológica?)
12	Logistically, do you think psychological therapy is accessible to you? (location, cost, time, etc.) (Logísticamente ¿Crees que la terapia psicológica es accesible para ti? (ubicación, costo, tiempo, etc.))
13	Have you used other methods to improve your mental health? (e.g., self-help books, meditation, talking with friends) Which ones? (¿Has usado otros métodos para mejorar tu salud mental? (por ejemplo, libros de autoayuda, meditación, hablar con amigos) ¿Cuáles?)
14	What do you think of these alternative methods compared to psychological therapy? (¿Qué opinas de estos métodos alternativos en comparación con la terapia psicológica?)

15	What changes would make you more open to seeking psychological therapy? (¿Qué cambios te harían estar más abierto a buscar terapia psicológica?)
16	Is there anything else you would like to share about your views on psychological therapy? (¿Hay algo más que te gustaría compartir sobre tus opiniones sobre la terapia psicológica?)

Interview Guide for Individuals Who Have Attended Therapy

Question number	Question
1	What was your opinion about psychological therapy before attending? (¿Cuál era tu opinión sobre la terapia psicológica antes de asistir?)
2	How long have you been attending psychological therapy? (pause for response) What led you to start? (¿Cuánto tiempo has estado asistiendo a terapia psicológica (pausa, para que la persona conteste), ¿qué te llevó a comenzar?)
3	What were your thoughts when beginning the therapeutic process? (¿Cuáles fueron tus pensamientos al momento de iniciar con el proceso terapéutico?)
4	Can you describe your experience with psychological therapy so far? (¿Puedes describir tu experiencia con la terapia psicológica hasta ahora?)
5	How has psychological therapy impacted on your mental health (pause for response) and overall well-being (then mention this part)? (¿Cómo ha impactado la terapia psicológica en tu salud mental (pausa, para que la persona conteste) y bienestar general (luego mencionar esta parte)?)
6	Did you face any challenges when considering starting therapy? What were they? How did they unfold? (¿Se presentó algún tipo de reto cuando consideraste iniciar la terapia psicológica? ¿Cuál/es? ¿Cómo fue/ron?)
7	What do you think your family and friends think about your decision to attend therapy? (¿Qué crees que opinan tu familia y amigos sobre tu decisión de asistir a terapia psicológica?)
8	Have you experienced any prejudice for attending psychological therapy? (¿Has experimentado algún prejuicio por asistir a terapia psicológica?)
9	What role do you think culture plays in your therapeutic process? (¿Cuál crees que sea el papel que juega la cultura en tu proceso terapéutico?)
10	Logistically, how accessible is psychological therapy for you? (location, cost, time, etc.) (Logísticamente ¿Qué tan accesible es la terapia psicológica para ti? (ubicación, costo, tiempo, etc.))
11	Have you faced any issues that made it difficult to continue attending therapy? (¿Has enfrentado algún problema que haya dificultado tu asistencia a terapia psicológica?)
12	What do you think are the main benefits of psychological therapy? (¿Cuáles crees que son los principales beneficios de la terapia psicológica?)
13	Is there any aspect of psychological therapy that you believe could be improved? (¿Hay algún aspecto de la terapia psicológica que consideras que podría mejorarse?)
14	Based on your experience, why do you think some people are unwilling to seek therapy? (Basado en tu experiencia, ¿por qué crees que algunas personas no están dispuestas a buscar terapia psicológica?)
15	What advice would you give to someone who is hesitant to seek psychological therapy? (¿Qué consejo darías a alguien que duda en buscar terapia psicológica?)

16	How would you describe your before-and-after experience with psychological therapy? (¿Cómo describirías tu antes y después de la terapia psicológica?)

Focus Group Interview Guide for Clinical Psychologists

Question number	Question
1	Can you describe the common reasons why people seek psychological therapy? (¿Puedes describir las razones comunes por las que las personas buscan terapia psicológica?)
2	What typical barriers prevent individuals from seeking psychological therapy? (¿Qué barreras típicas impiden que las personas busquen terapia psicológica?)
3	In your experience, what fears or concerns do clients usually have when starting therapy? (En tu experiencia, ¿cuáles son los miedos o preocupaciones que los clientes suelen tener al empezar la terapia psicológica?)
4	How important is the first session in establishing an effective therapeutic relationship? (¿Qué importancia tiene la primera sesión en establecer una relación terapéutica efectiva?)
5	What role do social and cultural factors play in clients' decisions to seek or avoid therapy? (¿Qué papel juegan los factores sociales y culturales en la decisión de los clientes de buscar o evitar la terapia psicológica?)
6	How do issues such as cost, location, and time affect clients' ability to attend therapy? (¿Cómo impactan temas como el costo, la ubicación y el tiempo en la capacidad de los clientes para asistir a terapia psicológica?)
7	Are there specific groups or demographics that face more significant barriers to accessing therapy? (¿Hay grupos o demografías particulares que enfrenten barreras más significativas para acceder a la terapia psicológica?)
8	How does social stigma affect people's willingness to seek psychological therapy? (¿Cómo impacta el estigma social en la disposición de las personas para buscar terapia psicológica?)
9	What other support systems do you recommend to clients? (e.g., support groups, community resources) (¿Qué otros sistemas de apoyo sugieres a los clientes? (por ejemplo, grupos de apoyo, recursos comunitarios))
10	Based on your professional experience, why do you think some people are reluctant to seek psychological therapy? (Basado en tu experiencia profesional, ¿por qué crees que algunas personas son reacias a buscar terapia psicológica?)
11	What do you think therapists can change or do to help normalize seeking psychological care? (¿Qué consideras que puedan los terapeutas cambiar o hacer para normalizar más la búsqueda de atención psicológica?)
12	Is there anything else you would like to share that might help better understand the reluctance to seek therapy? (¿Hay algo más que te gustaría compartir que pueda ayudar a entender la reticiencia a buscar terapia psicológica?).
